# Egg Consumption: Trends Over 48 Years, Patterns Across the Lifespan, and Predictors of Intake

**DOI:** 10.3390/nu17030533

**Published:** 2025-01-31

**Authors:** Donna Kritz-Silverstein, Ricki Bettencourt

**Affiliations:** 1 Herbert Wertheim School of Public Health and Longevity Science, University of California San Diego, La Jolla, CA 92093-0725, USA; 2 Department of Family Medicine, School of Medicine, University of California San Diego, La Jolla, CA 92093-0725, USA; 3 Division of Gastroenterology and Hepatology, School of Medicine, University of California San Diego, La Jolla, CA 92093-0725, USA; rbettencourt@health.ucsd.edu

**Keywords:** barriers, egg consumption, egg intake, patterns of intake, trends, lifespan

## Abstract

**Background/Objectives:** Eggs are an excellent nutritional source. However, historical associations of dietary cholesterol with serum cholesterol and cardiovascular disease, along with restrictive dietary guidelines may have been barriers to egg consumption. This study examines trends over time, patterns, and predictors of egg consumption in individuals followed for 48 years, and current barriers to usage. **Methods**: Participants were 6326 men and women enrolled in the Rancho Bernardo Study in 1972–1974 when asked about the number of eggs consumed/week. Subsequent egg intake was collected with food frequency questionnaires during clinic visits in 1988–1991 (*n* = 1627) and 1992–1996 (*n* = 1385), and with the original question on a 2021 mailed survey (*n* = 710), when barriers to intake were also queried. **Results**: The mean (±SD) number of eggs consumed was 3.6 ± 3.0 in 1972–1974, 1.8 ± 2.1 in 1988–1991, 1.8 ± 2.2 in 1992–1996, and 3.4 ± 3.5 in 2021. Comparisons within 5-year categories of enrollment age (<20, 20–24, 25–29, 30–34, 35–39, >40) showed no differences in egg intake between 1972 and 1974, and when older in 2021. Men consumed more eggs than women at all timepoints (*p*’s < 0.0001). High cholesterol and taking cholesterol-lowering medication were associated with lower egg consumption in 1972–1974 (*p*’s < 0.0001), but were not associated in 2021. Over 22% reported limiting egg intake in 2021; the majority cited cholesterol content of eggs or having high cholesterol as barriers. **Conclusions**: Egg consumption was responsive to dietary guidelines, decreasing over time then increasing by 2021 to levels similar to those of the early 1970s. Despite the abandonment of these guidelines, they continue to have a negative impact for a segment of society, suggesting the need for additional education on the health benefits of eggs.

## 1. Introduction

Eggs are an excellent source of protein and amino acids, as well as vitamins, nutrients, and bioactive compounds important for health, such as choline and the carotenoids, lutein, and zeaxanthin [1,2,3,4]. Although eggs are low in saturated fat, they contain high levels of dietary cholesterol [1,2]. Because it was thought that dietary cholesterol could raise serum cholesterol levels, thereby increasing the risk of CVD morbidity and mortality, guidelines were developed in 1968 by the American Heart Association that recommended limiting dietary cholesterol intake to less than 300 mg/day and no more than three whole eggs per week [5]. The recommended amount was further reduced to 200 mg/day in 2001 for those at high risk of heart disease [6]. Although a recent meta-analysis reported a significant increase in CVD risk and all-cause mortality with dietary cholesterol and egg consumption [7], most studies and meta-analyses showed that egg and dietary cholesterol intake have at most, only a negligible impact on serum cholesterol levels for most people [8,9,10]. Current (2015–2020) dietary guidelines for Americans do not specify a limit for dietary cholesterol [11].

The historical association of dietary cholesterol with serum cholesterol and, thus, CVD risk, along with the guidelines, may have served as a barrier to egg consumption. Studies using 1988–1994 [12], 2001–2008 [4], and 2001–2012 [13] data from the National Health and Nutritional Examination Survey (NHANES) all show similar results—that only about 20% of US adults consumed eggs on any given day and mean egg consumption was 24 g/day (1 egg = approximately 50 g). Comparisons by demographic characteristics showed that women had somewhat lower odds of consuming eggs than men [13]. Egg intake also varied by age. Compared to those aged 20–30 years, those aged 51–70 years had the greatest odds of consuming eggs followed by those aged 71 and older, although there were no differences in mean eggs consumed per day by age. Likewise, there were no differences in egg consumption by income, education, or food security status [13].

Only one previous study examined trends and patterns of egg consumption, but that was over a relatively short period of 12 years [13]. To our knowledge, no previous studies have examined trends over time and patterns in egg consumption across the lifespan, or predictors of egg intake in adults over a period spanning from a time shortly after egg intake guidelines were first introduced to a period after these guidelines were abandoned. Additionally, no studies have examined the extent to which perceptions concerning the cholesterol content of eggs and its impact on serum cholesterol persist as barriers to egg consumption.

The purpose of this study is to examine egg consumption trends over time, patterns of use across the lifespan, and predictors of intake in a cohort followed for 48 years. A secondary purpose was to examine current perceived barriers to egg consumption in older adults.

## 2. Materials and Methods

### 2.1. Participants

Data for this analysis comes from participants in the Rancho Bernardo Study. In 1972–1974, 6339 individuals representing 82% of all residents of the southern California community of Rancho Bernardo, were enrolled in a study of heart disease risk factors. This cohort has been followed with periodic research clinic visits and almost yearly mailed surveys through 2021. This study focuses on 6326 individuals who had egg intake assessed at 1972–1974, of whom 1627 also had egg intake assessed at a 1988–1991 clinic visit, 1385 had egg intake assessed at a 1992–1996 clinic visit, and 710 had egg intake assessed with a mailed survey in 2021 along with barriers to intake.

This study and all previous clinic visits were approved by the Human Research Protections Program of the University of California San Diego (IRB #191902). All individuals provided written informed consent prior to participation.

### 2.2. Egg Consumption

Egg consumption was assessed at enrollment in 1972–1974 with a single question on a self-administered survey. Specifically, participants were asked to write the answer to the question, “How many eggs do you eat per week? (visible eggs only)”. At the 1988–1991 and the 1992–1996 visits, egg consumption was reassessed with the Willet Food Frequency Questionnaire (FFQ) [14]. The FFQ provides participants with a list of 153 foods including eggs; for each food, closed-ended questions ask them to indicate the frequency of consumption over the past year and the usual portion size consumed. Response choices for egg consumption were never, 1–3/month, 1/week, 2–4/week, 5–6/week, 1/day, 2–3/day, 4–5/day, and 6+/day. Due to a low response frequency, the highest categories of egg intake were combined into a single category of ≥5/week. In 2021 a mailed survey was sent to surviving participants which included the original open-ended question about egg consumption. To assess barriers to egg consumption, participants were asked about whether they limited their egg intake; those who responded affirmatively were asked to give the reasons for limiting egg intake.

### 2.3. Covariates

Self-administered surveys were used at all clinic visits to obtain information on demographic characteristics such as age and education (high school or less vs. some college or more), as well as physician diagnosis of diseases including diabetes (no/yes) and high cholesterol (no/yes), as well as the use of antidiabetic (no/yes) and cholesterol-lowering medication (no/yes). The 2021 mailed survey also collected this information.

### 2.4. Statistical Analysis

The original question used to assess egg intake in 1972–1974 and subsequently used in 2021 was open-ended, whereas the FFQ provided response choices. Because of this non-comparability and to be able to examine patterns and trends over time, it was necessary to harmonize responses on the FFQ with those on the open-ended question. To achieve this, response choices on the FFQ with a range were re-coded to the midpoint of the range (so that the error would be random); responses providing a number per day were multiplied by 7 to obtain the number of eggs per week; responses providing a number per month were divided by 4 to obtain the number of eggs per week; responses of never and responses providing number per week did not change. Comparisons of egg consumption between men and women at the four different time points (1972–1974, 1988–1991, 1992–1996, and 2021) were performed with independent *t*-tests. Comparisons of egg intake in 1972–1974 and 2021 by 5-year categories of age at enrollment (1972–1974), were performed with signed rank tests. Multiple linear regression analyses were used to examine the association of several predictors (age, sex, education, diabetes, use of medication for diabetes, high cholesterol, and the use of cholesterol-lowering medication) with egg intake in 1972–1974, and separately, in 2021. Because more than one reason could be given for limiting egg intake in the 2021 survey, a multiple-response variable was created to examine reasons for restricting egg consumption. Sensitivity analyses to examine survival bias compared the enrollment characteristics of those who did and did not contribute data in 2021 using independent *t*-tests, Wilcoxon, and chi-square analysis.

## 3. Results

### 3.1. Demographic and Other Characteristics

The average duration of time elapsed between enrollment and last contact date in 2021 was 47.8 ± 0.6 years. Demographic and other characteristics at each time point, when egg intake was assessed, are shown in Table 1. Of interest to note is that in 1972–1974, only 3.7% reported a diagnosis of diabetes and 13.3% reported having high cholesterol. Those rates rose to 39.7% for diabetes and 74.7% for high cholesterol in 2021. Likewise, reported use rose from 2.2% for antidiabetic medications and 2.6% for cholesterol-lowering medications in 1972–1974, to 36.0% and 70.7%, respectively, in 2021.

### 3.2. Egg Consumption Trends over Time

**Figure 1:**
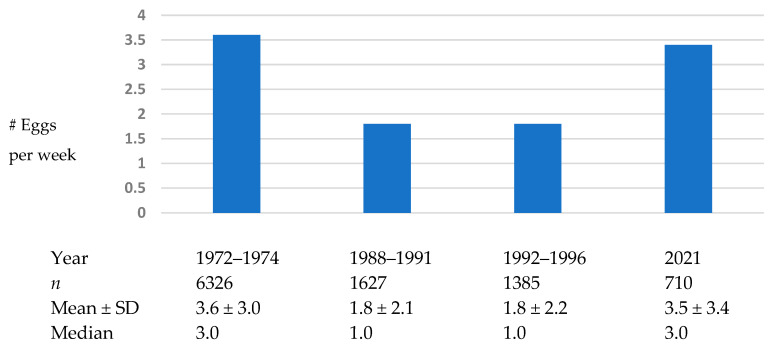
Distributions of number of eggs consumed per week in 1972–1974, 1988–1991, 1992–1996, and 2021; Rancho Bernardo, CA.

Figure 1 shows the distributions of number of eggs consumed per week in 1972–1974, 1988–1991, 1992–1996, and 2021. As shown, mean egg consumption in 1972–1974 was 3.6 ± 3.0 per week. Mean egg consumption dipped to 1.8 per week in 1988–1991 and 1992–1996. However, in 2021, mean egg consumption was 3.5 per week, similar to the level it was in 1972–1974.

**Figure 2:**
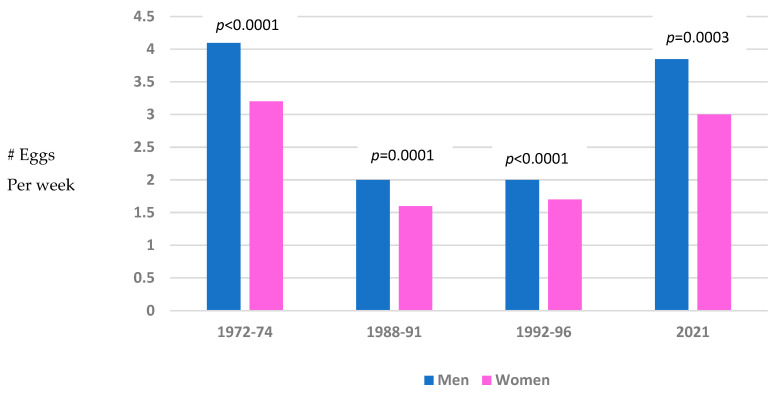
Mean egg intake in men vs. women at each time point; Rancho Bernardo, CA.

There were significant sex differences in egg consumption per week (Figure 2). At all time-points over the 48 years examined, men consumed significantly more eggs per week than women (means respectively = 4.1 vs. 3.2, *p* < 0.0001 in 1972–1974; 2.0 vs. 1.6, *p* = 0.0001 in 1988–1991; 2.0 vs. 1.7, *p* < 0.0001 in 1992–1996; and 4.1 vs. 3.0, *p* = 0.0003 in 2021).

### 3.3. Patterns of Use Across the Lifespan

**Figure 3:**
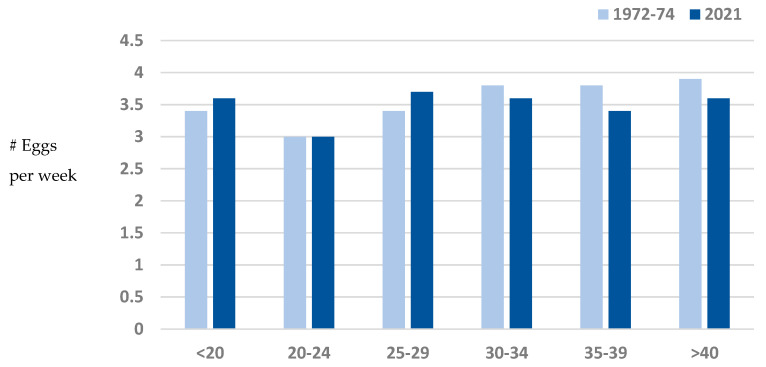
Egg intake in 1972–1974 vs. 2021 by 5-year categories of enrollment age; Rancho Bernardo, CA.

To examine patterns of use across the lifespan, mean egg intake per week in 1972–1974 was compared to egg intake per week in 2021 within each 5-year category of enrollment age among those who responded at both time points. As shown in Figure 3, within those aged <20, 20–24, 25–29, 30–34, 35–39, and >40 at enrollment, there were no differences between egg consumption in 1972–1974 and egg consumption in 2021 (*p*’s > 0.10). Egg intake among those aged 20–24 at enrollment was slightly lower (about 0.5 eggs/per week) than those in the other age categories, and that difference remained in 2021.

### 3.4. Predictors of Egg Intake

Multiple regression analysis was used to examine the predictors of egg consumption at each time point (Table 2). As shown, female gender was predictive of lower egg intake regardless of the year when data were collected. In 1972–1974 high cholesterol and the use of cholesterol-lowering medication were associated with lower egg consumption (*p*’s < 0.0001). Similarly, in 1992–1996, the use of cholesterol-lowering medication was significantly associated with lower egg consumption (*p* = 0.0005). However, in 2021, both high cholesterol and the use of cholesterol-lowering medication were no longer associated with the number of eggs consumed per week. The diagnosis of diabetes or the use of anti-diabetic medication was positively associated with egg intake at enrollment (*p*’s < 0.0001), but not at subsequent visits. Additionally, age was not associated with egg consumption at any time point, and education had a small positive association in 1972–1974, a small negative association in 1992–1996, but was not associated with either 1988–1991 or 2021.

### 3.5. Perceived Barriers to Egg Consumption

There were 157 individuals (22.1%) who reported limiting their intake of eggs on the 2021 survey. Of these, only 1.3% reported limiting intake because of being allergic to eggs, and 6.4% reported not liking the taste. Overall, 21.0% reported limiting egg intake because they thought it was healthier, 10.2% mentioned the high cholesterol content of eggs, and 25.5% gave multiple reasons, usually including either the high cholesterol content of eggs, or that it was a doctor’s recommendation because of their high cholesterol level, or both of these reasons. Only 18.5% said there was no particular reason.

### 3.6. Sensitivity Analyses

Sensitivity analyses examining the potential for survivor bias compared the enrollment characteristics of those who did and did not contribute data in 2021. Compared to those who contributed data at both time points, those who did not contribute data in the 2021 survey were younger (*p* < 0.001), more likely to be male (*p* < 0.001), had lower rates of college education (*p* < 0.001), and higher rates of diabetes (*p* = 0.007), high cholesterol (*p* = 0.001), and cholesterol-lowering medication use (*p* < 0.001), but consumed somewhat fewer eggs per week (3.9 vs. 3.4, *p* = 0.02) at enrollment.

## 4. Discussion

### 4.1. Study Outcomes

Guidelines limiting egg consumption and dietary cholesterol intake were first promoted by the American Heart Association in 1968 [5], and then widely adopted by other agencies in 1995 [15]. It was not until 2015 that this recommendation was abandoned. Results of this novel study with 48 years of follow-up, showed that egg consumption by these community-dwelling men and women was responsive to the changing dietary guidelines regarding dietary cholesterol intake. Intake was higher in 1972–1974 with a mean of 3.6 eggs per week. This decreased to 1.8 eggs in data from the 1988–1991 and 1992–1996 visits, and increased to 3.5 eggs per week in 2021, an amount similar to that observed in 1972–1974. Among participants who were young or middle-aged at enrollment, within each 5-year age category, egg intake patterns were similar 48 years later, when older, and after recommendations were abandoned. This suggests that without the influence of dietary recommendations, patterns of egg consumption may be fairly stable across the lifespan. To our knowledge, this is the first and only study to examine egg consumption over such a long period of time and to capture the impact of the changing guidelines over almost half a century.

The results of this study are in contrast to the single previous study that examined trends in egg consumption across time and reported that egg consumption was unchanged from 2001 to 2012 [13]. However, while that study used the nationally representative NHANES data, in contrast to the present study, its follow-up was limited to 12 years and spanned over a period of time that did not include major changes in the guidelines surrounding egg intake [13].

In both the NHANES study [13] and this study, several demographic factors in relation to egg consumption were examined. In this study, men consumed significantly more eggs per week than women at each time point. Although we can offer no explanation for this sex difference, it is in accord with the NHANES study, which also found that women consumed fewer eggs per week than men [13]. In this study, there was no association between age and the number of eggs consumed per week. This is in accord with results from the NHANES study [13], which reported no differences by age in mean eggs consumed per day. That study also reported that older individuals had greater odds of consuming any eggs as compared to those aged 20–30 years. Similarly, in the present study, there was slightly lower egg intake among those aged 20–24 at enrollment, which was also evident in 2021. In this study, education had small, inconsistent associations with egg consumption at earlier time points, but no association in 2021. This is also in accord with the lack of association between education and the odds of any egg intake in the 2001–2012 NHANES data [13].

In the present study, prevalence rates of high cholesterol increased from 13.3% to 74.7%, and the use of cholesterol-lowering medication increased from 2.6 to 79.7% between 1972 and 74 and 2021, likely reflecting the changing definitions of hypercholesterolemia and the widespread use of statins. Additionally, in this study, self-reported diagnosis of high cholesterol and use of cholesterol-lowering medication were each associated with lower egg consumption in 1972–1974. However, by 2021, these variables were no longer predictive of egg consumption, possibly reflecting the increase in knowledge of the lack of association between dietary cholesterol and serum cholesterol.

Eggs contain high levels of vitamins, nutrients, and amino acids, as well as protein. However, unlike other sources of protein, they are also low in fat [1,2,3,4]. Combined with their relatively low cost, eggs may be of particular importance for older individuals. However, the results of this study suggest that the earlier misinformation about dietary cholesterol in eggs and the inaccuracies perpetuated by inappropriate guidelines still exist and create barriers to consumption. For instance, in this study, over a fifth of the participants reported limiting egg consumption, with the majority indicating doing so either because of the high cholesterol content of eggs, because a doctor recommended it due to their high cholesterol, or because they thought it was healthier.

No previous study has examined whether the historical link of dietary cholesterol with heart disease influences current behavior. This study, with data collected 6 years after the abandonment of guidelines restricting egg intake, suggests that the misinformation related to egg intake continues to affect perceptions and ultimately behavior. This misinformation, whether concerning the association of egg intake with cardiovascular disease, or the influence of dietary cholesterol on serum cholesterol, serves as a barrier to egg consumption [15], and outdated information can still be found on some health-related websites [16]. Increased education for physicians and other health professionals, as well as better marketing to the public concerning the benefits and cost-effectiveness of eggs, is warranted.

### 4.2. Limitations and Strengths

Several potential limitations of this study were evaluated. Egg intake was based on self-reports, which may be subject to recall bias. However, others point out that self-reported dietary data may provide information on food intake, behaviors, and eating patterns that cannot be obtained via lab assessment of biomarkers, and that self-reported data can be useful for examining the associations between diet and disease [17]. Only whole eggs were assessed and eggs used in cooking other dishes were not counted. Thus, results may underestimate the true patterns and changes over time.

Members of the Rancho Bernardo Cohort are largely white, middle-class, relatively well-educated, and have little difficulty accessing medical care. On the one hand, this homogeneity may limit the generalizability of the results. However, on the other hand, this homogeneity may be advantageous in that results are less likely to be confounded due to differences in education or the ability to afford medical care. Additionally, previous comparisons with other large, nationally representative cohorts showed that while participants in the Rancho Bernardo Study have a somewhat lower rate of obesity [18], they are similar in behaviors, such as smoking [19] and alcohol use [20], as well as rates of chronic diseases, such as diabetes and impaired glucose tolerance, and clinical measures, such as blood pressure and total cholesterol [21,22]. Given that all participants were US residents, we cannot exclude the possibility that the results of this study may not generalize to those residing in other countries or from other cultures where egg consumption may be different.

Although a large sample was originally enrolled in this study, smaller subsamples participated in the follow-up, and not every individual participated in each follow-up. This was due to later clinic visits concentrating only on individuals older than age 50 who would be more susceptible to the chronic diseases that were the focus of follow-up visits. Additionally, as with other studies of older individuals, attrition due to death and illness also reduced the follow-up sample size. Given the long follow-up, only about 25% of the initially enrolled cohort was still alive at the time of the 2021 survey. We cannot exclude the possibility of survivor bias, whereby only the healthiest people may have survived to contribute data to this study. Sensitivity analyses showed that those who did not contribute data in 2021, were younger, less educated, more likely to be male, had more illnesses, and consumed fewer eggs per week at enrollment. These differences could have affected the trends and patterns observed here.

Because egg consumption was assessed at only four points in time, we cannot determine the precise timing at which guidelines surrounding egg intake were adopted or abandoned. However, given that the adoption of guidelines by the general public likely takes some time from when they are first put forth by an agency, it is reasonable that the 1972–1974 assessment was close to the time when individuals first became aware of them. Although egg intake in 2021 was higher and similar to that in 1972–1974, we do not know exactly when individuals abandoned the guidelines.

This study also had several strengths that should be mentioned, including a relatively large sample size, and a duration of follow-up that was almost half a century and far exceeds that of other studies. Additionally, by examining participants’ perceived barriers to egg consumption, we were able to determine that the majority of the 22.1% who limit egg intake do so because of the continued misperception that the high cholesterol content of eggs is unhealthy, and that egg intake should be limited if one has high cholesterol levels.

## 5. Conclusions

This novel study followed the behavior of individuals over 48 years—far longer than any other study. Because of this, we were able to show that guidelines on egg consumption, whether appropriate or not, impacted egg intake. Trends in egg consumption over almost half a century, in this community-dwelling cohort mirrored the changing dietary guidelines, decreasing over time and then increasing by 2021 to levels similar to those of the early 1970s. Among those young and middle-aged at enrollment, egg intake was similar 48 years later, when older, suggesting that without dietary recommendations, egg intake patterns might be stable over the lifespan. Cholesterol levels and the use of cholesterol-lowering medication were no longer associated with egg intake in 2021, mirroring the abandonment of the dietary guidelines and increasing knowledge of the lack of association found in research. However, despite the abandonment of these dietary guidelines, they continue to serve as a barrier and negatively impact egg consumption for a segment of society. This suggests that to counter the lingering effects of outdated dietary guidelines, additional education for physicians, other health professionals, and the general public, as well as targeted campaigns among older individuals regarding the importance of eggs for the maintenance of health are needed.

## Figures and Tables

**Table 1:** Demographic and other characteristics of participants in 1972–1974, 1988–1991, 1992–1996, and 2021; Rancho Bernardo, CA.

	1972–1974			1988–1991			1992–1996			2021		
Characteristic	*n*	Mean	SD	*n*	Mean	SD	*n*	Mean	SD	*n*	Mean	SD
Age (years)	6326	52.7	19.7	1627	71.1	10.7	1385	71.0	11.5	710	74.0	10.4
	%			%			%			%		
Male	46.5			42.6			39.4			44.7		
Some college or more	60.3			70.9			74.5			51.5		
Diabetes	3.7			6.4			5.7			39.7		
High cholesterol	13.3			N/A			N/A			74.7		
Antidiabetic medication	2.2			N/A			2.2			36.0		
Cholesterol-lowering meds	2.6			N/A			11.9			70.7		

N/A = not available; data not collected during that visit.

**Table 2:** Association of characteristics with egg intake at each time point; Rancho Bernardo, CA.

	1972–1974		1988–1991		1992–1996		2021	
Characteristic	Beta	*p*-Value	Beta	*p*-Value	Beta	*p*-Value	Beta	*p*-Value
Age (years)	−0.001	0.46	0.004	0.44	0.008	0.12	−0.001	0.97
Female gender	−0.96	**<0.0001**	−0.40	**0.0001**	−0.33	**0.0066**	−1.11	**<0.0001**
Some college or more	0.41	**<0.0001**	0.11	0.33	−0.33	**0.0163**	0.04	0.85
Diabetes	0.77	**<0.0001**	0.27	0.19	0.13	0.62	1.05	0.09
High cholesterol	−1.00	**<0.0001**	NA	--	NA	--	−0.49	0.20
Anti-diabetic medication	1.28	**<0.0001**	NA	--	0.40	0.32	0.76	0.23
Cholesterol-lowering meds	−1.53	**<0.0001**	NA	--	−0.64	**0.0005**	−0.47	0.23

N/A = not available; data not collected during that visit.

## Data Availability

Data for this analytic study are from the publicly archived Rancho Bernardo Study and are available at https://knit.ucsd.edu/ranchobernardostudy/ (accessed 2 October 2021).

## Abbreviations

The following abbreviations are used in this manuscript:

CVD	Cardiovascular Disease
FFQ	Food Frequency Questionnaire
